# Quality of oxytocin and misoprostol in health facilities of Rwanda

**DOI:** 10.1371/journal.pone.0245054

**Published:** 2021-01-08

**Authors:** Thomas Bizimana, Nhomsai Hagen, Gesa Gnegel, Pierre Claver Kayumba, Lutz Heide

**Affiliations:** 1 Department of Pharmacy, School of Medicine and Pharmacy, College of Medicine and Health Sciences (CMHS), University of Rwanda, Kigali, Rwanda; 2 Pharmaceutical Institute, Eberhard Karls University Tübingen, Tübingen, Germany; 3 East African Community Regional Centre of Excellence for Vaccines, Immunizations and Health Supply Chain Management (EAC RCE-VIHSCM), University of Rwanda, Kigali, Rwanda; Institute of medical research and medicinal plant studies, CAMEROON

## Abstract

Sustainable Development Goal 3.1 calls for a reduction of the maternal mortality ratio to less than 70 per 100,000 live births by 2030. The most important cause of maternal mortality is post-partum haemorrhage (PPH). Oxytocin injections and misoprostol tablets are medicines of first choice for the management of PPH in low- and middle-income countries (LMICs). Unfortunately, both substances are chemically unstable, and previous studies have revealed serious quality problems of these medicines in LMICs. The present study is the first report on their quality in Rwanda. From 40 randomly selected health facilities (hospitals, health centers, retail pharmacies and private clinics) in different parts of Rwanda, as well as from six wholesalers and government stores, oxytocin injections and misoprostol tablets were collected. Oxytocin storage temperatures in the health facilities were monitored for six months using temperature data loggers, and found to correctly follow the storage requirements stated by the manufacturers (2–8°C, or room temperature) with few minor deviations. Oxytocin injections (57 samples, representing seven batches of four brands) were tested for their oxytocin content and pH value according to the United States Pharmacopeia. Twenty-four samples from three European manufacturers passed all tests. However, all nine samples of one batch of a Chinese manufacturer showed an excessive content of oxytocin (range 117.2–121.5% of the declared amount). Another batch of the same manufacturer showed extreme variations of the concentration of the preservative benzyl alcohol. Misoprostol tablets (25 samples, representing ten batches of six brands) were tested for content and dissolution according to the International Pharmacopoeia. Fifteen samples passed, but all 10 samples of two brands from India failed with extreme deviations, containing only 42.5–48.7% of the stated amount of misoprostol. In conclusion, oxytocin quality in Rwanda was better than reported from other African countries. However, two extremely substandard brands of misoprostol tablets were found. The Rwandan authorities reacted quickly and efficiently, and recalled these substandard medicines from the market. For oxytocin and misoprostol, with their well-known problems of quality and stability, procurement should possibly be restricted to medicines which are WHO-prequalified or which have been manufactured in countries with stringent regulatory authorities.

## Introduction

In the year 2017, an estimated number of 295,000 women around the world died due to complications of pregnancy and childbirth [1]. The highest maternal mortality ratio is observed in the sub-Saharan region of Africa [1, 2]. Post-partum hemorrhage (PPH) is the most important cause of maternal mortality, and 50% of all cases of PPH worldwide occur in Africa [3]. Rwanda, a low-income country in sub-Saharan Africa [4], has successfully lowered its maternal mortality ratio from 1160 down to 248 per 100,000 live births in the years from 2000 to 2017 [1]. Efforts are made by the government of Rwanda to further reduce the maternal mortality ratio to less than 70 per 100,000 live births by 2030, in accordance with the Sustainable Development Goal (SDG) 3.1 [5, 6]. To achieve this target, oxytocic medicines which are used to treat and prevent PPH are of principal importance. Oxytocin injections and misoprostol tablets are among the medicines of first choice for the prevention and treatment of PPH [3, 7]. They are included as oxytocics (uterotonics) in the WHO model list of essential medicines [8] and in the Rwanda National List of Essential Medicines for adults [9]. Also, they have been included in the list of 13 life-saving items prepared by the United Nations Commission on Life-Saving Commodities for Women and Children (UNCoLSC) [10], as the only medicines for the management of PPH.

The use of substandard and falsified medicines has been shown to result in serious public health problems [11]. Especially in LMICs, the quality of medicines often fails to meet the pharmacopeial specifications, and this has far-reaching adverse consequences for patients, families, national health systems and the economy [11, 12]. The use of substandard oxytocin or misoprostol preparations in the management of PPH may lead to therapeutic failure in the treatment of excessive bleeding, and even to the death of the patient [13, 14]. Avoiding such preventable deaths is one of the key measures required to reach SDG 3.1.

**Fig 1:**
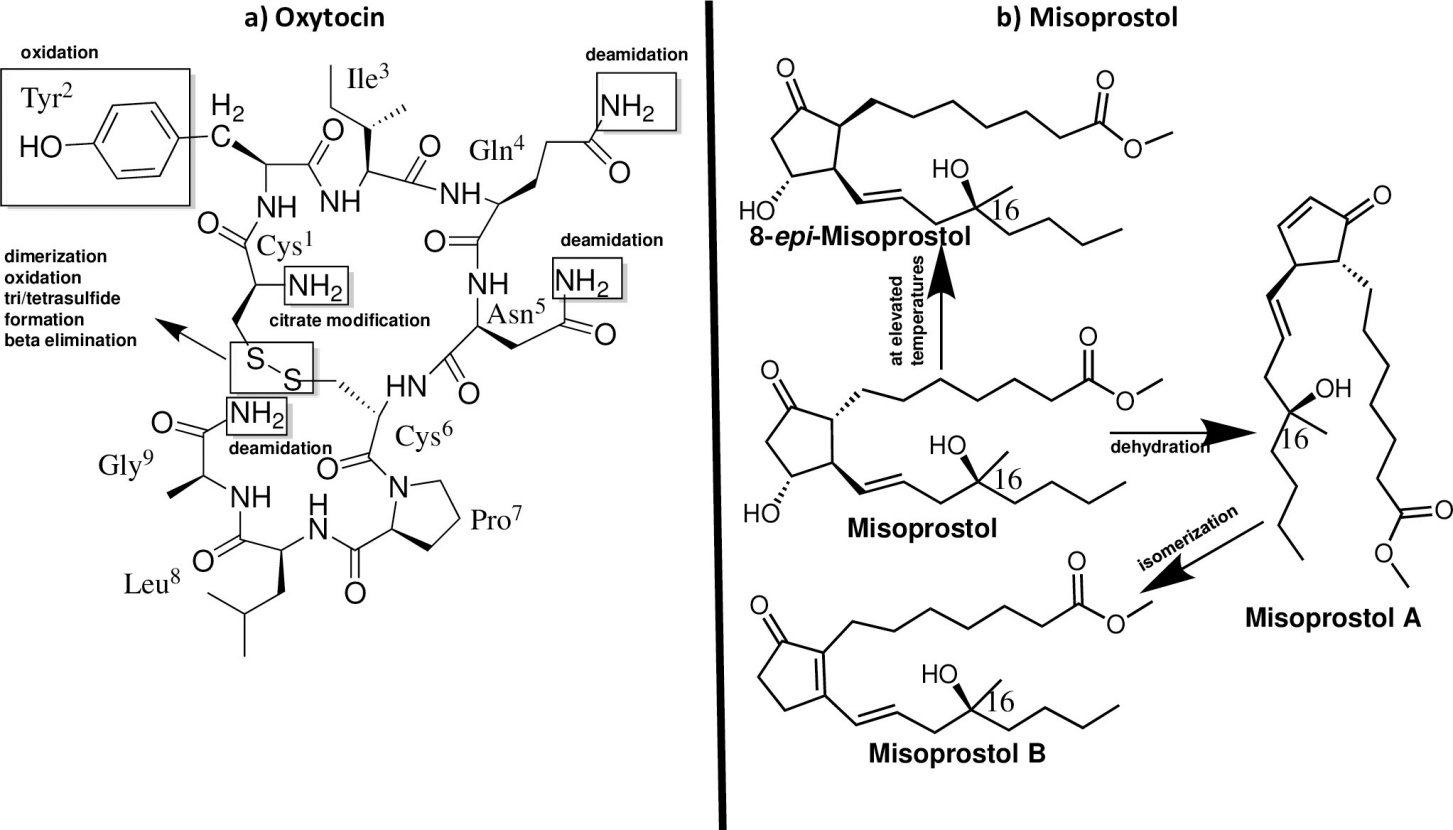
Structures of oxytocin (modified from [21]) and misoprostol, and their typical degradation mechanisms. Commercial misoprostol is a mixture, containing the depicted structure, its epimer at C16, and the enantiomers of both compounds. Likewise, the degradation products of misoprostol contain the corresponding stereoisomers.

Unfortunately, oxytocics are sensitive to environmental conditions. Oxytocin itself is a peptide hormone containing nine amino acids with an intramolecular disulfide bridge (Fig 1) [15]. It is highly sensitive to elevated temperatures and may degrade quickly when inappropriately stored, especially in tropical climates [16, 17]. The World Health Organization (WHO) recommends to store all preparations of oxytocin in the refrigerator, i.e. between 2°C and 8°C [16]. However, some commercial oxytocin preparations carry recommendations for non-refrigerated storage [18, 19]. Oxytocin stability furthermore depends on the pH value, with an optimum stability at pH 4.5 [20]. Both the United States Pharmacopeia (USP) and the International Pharmacopeia (Ph. Int.) demand that oxytocin injections must have a pH value between 3.0 and 5.0 [19].

A systematic review published by Torloni et al. in 2016 [13] listed eight studies on the quality of oxytocin conducted in LMICs. In a subsequent systematic review published by the same authors in 2020 [14], the number of included oxytocin quality studies had increased to 14. Overall, 39.7% of the oxytocin samples investigated in all these studies had been reported to fail quality testing. An insufficient content of the active pharmaceutical ingredient (API) represented by far the most frequently found deficiency. The overall percentage of failing samples had been 31.4% for studies conducted in the time period of 2000–2011 (n = 363 samples), but had increased to 44.4% for studies conducted from the year 2012 onwards (n = 611 samples) [14]. This indicates that in the last two decades, the quality problems of oxytocin have increased rather than decreased.

The percentage of oxytocin samples reported to fail quality testing varies notably between different studies. Anyakora et al. [22] reported that 74.2% of the 159 oxytocin samples collected in Nigeria failed quality testing. Similarly, Stanton et al. [23] reported a failure rate of 73.9% upon investigation of 46 oxytocin samples from Ghana. On the other hand, Hagen et al. [24] showed that only 11% out of 65 oxytocin samples collected in Malawi failed testing, and only with moderate deviations from the pharmacopeial specifications. Another study conducted in Ethiopia reported a failure rate of only 4% within 45 oxytocin samples [25]. In all these studies, the failure rate resulted nearly exclusively from an incorrect API amount determined in the samples [14]. Therefore, the different failure rates reported are not due to different parameters being tested in the different studies.

Misoprostol (Fig 1) is an analog of prostaglandin E1. Commercial preparations contain a mixture of both its epimers at C-16, and their enantiomers [26]. Misoprostol is a viscous oil at room temperature and is extremely unstable in the presence of water [27]. Both the raw material and the finished products have to be carefully protected from humidity [27, 28]. In finished pharmaceutical products, misoprostol must be stabilized in form of a 1% dispersion in hydroxypropyl methylcellulose (HPMC) [29], since in the absence of HPMC misoprostol quickly undergoes dehydration, isomerization and epimerization reactions (Fig 1), resulting in a loss of activity. A study by Hall [28] on 215 misoprostol samples, collected in 15 LMICs, reported an incorrect API content in 45% of the samples, and a decomposition of misoprostol in those samples which were packaged in plastic-aluminium blisters. Therefore, it has been strongly recommended that misoprostol tablets should be packaged in double-sided aluminium blisters to protect them from moisture [28]. Storage of misoprostol tablets outside the blisters exposes them to moisture and has been shown to quickly decrease the amount of the active ingredient, and also to reduce hardness and increase friability of the tablets [27]. In spite of these well-documented problems, misoprostol quality in LMICs has received much less attention than oxytocin quality. The above-mentioned review published by Torloni et al. [14] lists, besides the study by Hall, only two further studies which investigated the quality of misoprostol tablets: Anyakora et al. [22] reported that 56 (33.7%) out of 166 misoprostol samples collected in Nigeria failed quality testing due to incorrect API content, but the study did not state the exact amount of API detected in the samples. Hagen et al. [24] reported that 5 (17%) out of 30 misoprostol samples from Malawi failed pharmacopeial specifications, notably all five with extreme deviations since they contained only 12.7–30.2% of the declared amount.

So far, no data on the quality of oxytocin injections and misoprostol tablets in Rwanda have been published, although the above-mentioned findings from other LMICs indicate that the presence of substandard preparations is likely. Therefore, in the present study samples of oxytocin injections and misoprostol tablets were collected from randomly selected government, faith-based and private health facilities and drug outlets, as well as from government medical stores and private wholesalers in Rwanda, and were investigated for their quality according to the United States Pharmacopeia (USP) and the International Pharmacopeia (Ph. Int.), respectively. In parallel to this study, an evaluation of the availability and prices of essential medicines in health facilities of Rwanda, also beyond oxytocin and misoprostol, has been carried out, and the results have been published elsewhere [30].

## Methods

### Study design and ethical approval

The study protocol and the methods for collection and investigation of the samples were designed following the MEDQUARG guidelines [31] and the WHO Guidelines on the Conduct of Surveys of the Quality of Medicines [32]. Ethical approval to conduct this study was obtained from the College of Medicine and Health Sciences Institutional Review Board (CMHS-IRB) of the University of Rwanda with approval notice No. 026 /CMHS IRB/2018. An authorization to access health facilities and to conduct this study was kindly granted by the Ministry of Health, Rwanda (reference No. 20/1361/DGPHFIS/2018). In fulfilment of the requirements for sample transfer from Rwanda to Germany, a Material Transfer Agreement (MTA) was signed between investigators and the Rwandan Ministry of Health. Consent to import medicine samples for analysis was also obtained from German authorities (Regierungspräsidium Tübingen, Leitstelle Arzneimittelüberwachung).

### Selection of sampling sites

Samples of oxytocin and misoprostol were collected in Kigali city and in five districts representing the provinces of Rwanda, i.e. Bugesera district (Eastern Province), Karongi district (Western Province), Musanze district (Northern Province), and Muhanga and Kamonyi districts (Southern Province). Government, faith-based and private facilities were included, i.e. government district hospitals and health centers, faith-based district hospitals and health centers, private retail pharmacies and private clinics/hospitals. A list of these health facilities in Kigali city and in the five selected districts was obtained from the Ministry of Health, comprising altogether 13 district hospitals (government or faith-based), 77 government health centers, 44 faith-based health centers, 36 private clinics, and 234 private retail pharmacies. For each of the five districts and for Kigali city, two hospitals and two health facilities of each of the other categories (government health centers; faith-based health centers; private retail pharmacies; private clinics) were randomly selected using the RAND function of Microsoft Excel. However, in each of the two districts Muhanga and Kamonyi only a single district hospital existed; these were included into the study. In Musanze district, no district hospital existed, but a government referral hospital, and this was included. Therefore, a total of 57 health facilities and private retail pharmacies were selected. In the course of the study visits, it turned out that one of the selected district hospitals was a specialized orthopedic hospital and did not stock oxytocin or misoprostol. Also, 11 of the 12 selected private clinics stated that they did not store these medicines, and 5 of the 12 retail pharmacies had none of these two medicines available. Therefore, oxytocin and/or misoprostol could be collected from 40 health facilities and retail pharmacies, and these are listed in S1 Table.

**Fig 2:**
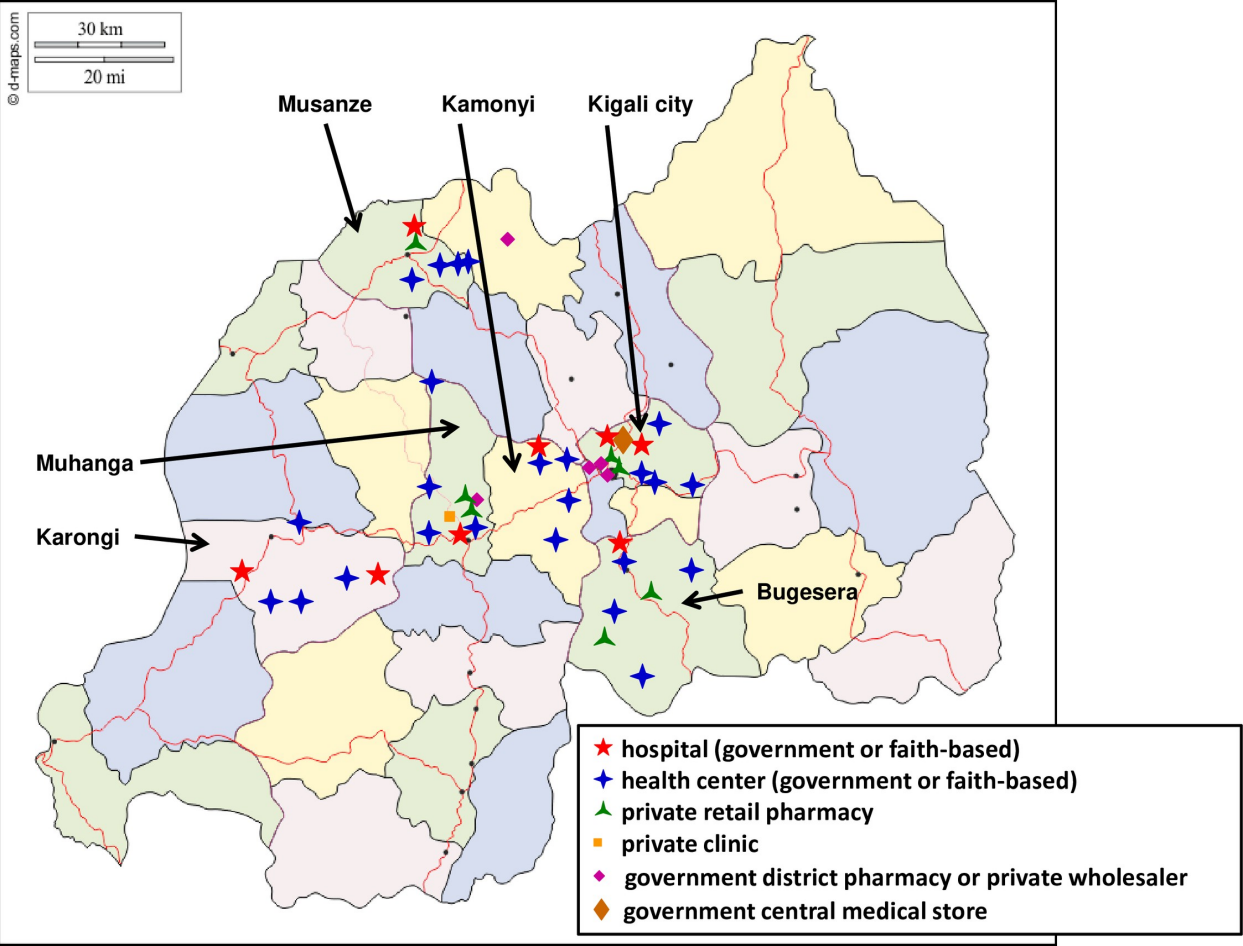
Map of the location of the 46 facilities from which oxytocics were collected. Reprinted (with modifications) from https://d-maps.com/conditions.php?lang=en under a CC BY license, with permission from d-maps.com, original copyright 2007–2020.

In addition to health facilities and retail pharmacies, oxytocics were also collected from the government central medical store (Medical Procurement and Production Division [MPPD]), from two government district pharmacies and from three large private wholesalers. Therefore, samples were collected from a total of 46 different facilities. Fig 2 shows the location of the facilities on a map of Rwanda.

In health facilities which stored oxytocics both in their pharmacy stores and in their maternity wards, samples were collected from both sites if these medicines were available there. Thereby in total samples were collected from 44 storage rooms and 20 maternity wards, as listed in S1 Table.

### Sample collection

Sampling was done by the investigator T.B. in March 2018 for the pilot study in Muhanga district, and in September-October 2018 for the main study in the other districts and Kigali city. At each sampling site, from every available brand and batch, 10 vials of oxytocin and 50 tablets of misoprostol were collected if available. A minimum amount of 5 vials of oxytocin and 10 tablets of misoprostol was collected.

When sampling from government and faith-based health facilities, the collected vials and tablets were replaced with medicines which had been purchased by the investigator from the government central medical store or two government district pharmacies, in order to avoid causing stock-outs in the health facilities by the sampling. Samples collected from private facilities (retail pharmacies, private clinics and wholesalers) were paid for in cash. In health facilities and retail pharmacies, samples were collected by an overt approach, i.e. the investigator informed the staff about the purpose of the visit, and the “Sample Site & Drug Purchase Records” shown in S1 Fig were filled and signed by the investigator and by the responsible person at the health facility. Samples from private wholesalers were obtained through a local retail pharmacy using a mystery shopper approach, and no “Sample Site & Drug Purchase Records” were prepared for these purchases.

Upon sample collection, each sample was immediately labeled with a unique sample code. All samples of oxytocin were transported to a central collection site in Rwanda in a 12 V plug-in refrigerator, and subsequently stored between 2°C and 8°C until shipment to Germany. Misoprostol tablets were transported and stored at a temperature not exceeding 25°C. Samples were hand-carried to Tübingen University, Germany, by the investigator T. B. on commercial passenger flights in May 2018 (for the pilot study) and in November 2018 (for the main study). They were placed into appropriate storage conditions at Tübingen University within 24 hours after departure from Rwanda. A temperature data logger was kept with the samples at all times to record the temperature during transport and intermediate storage.

### Assessment of oxytocin storage temperatures

Oxytocin storage temperatures were recorded using temperature data loggers (Tempmate M1 by imec Messtechnik GmbH, Heilbronn, Germany). These were placed by the investigator at the storage sites of oxytocin at the time of sample collection, and they automatically recorded the temperature every 10 minutes. Private wholesalers were contacted by a mystery shopper approach, and therefore no temperature data logger could be placed. Temperature data loggers were recollected by the investigator after six months, and the mean kinetic temperature (MKT) was calculated by the imec Messtechnik software.

### Registration status of collected medicines

To enquire the registration status of the medicines collected in Rwanda, the Rwanda Food and Drug Authority (RFDA) was contacted. However, the full registration process of medicines by RFDA had not yet been in effect at the time of sample collection. It could not be established which of the collected preparations had been pre-registered according to the previous procedures.

### Packaging examination

The information stated on the packaging and in the package inserts of the samples were examined visually for the presence of irregularities or inconsistencies, such as spelling mistakes, unusual batch numbers, unexpected or modified manufacturing or expiry dates, or signs of repacking. The individual dosage units (oxytocin vials and misoprostol tablets) were inspected for visible deficiencies, like color changes, suspended particles within vials, etc. In addition, for misoprostol tablets the material of the primary packaging was recorded, as this is important for misoprostol stability [28].

### HPLC analysis

Oxytocin injections were analyzed for identity, assay and pH value following the respective monograph of the United States Pharmacopoeia 40 (USP 40). The assay was performed using High Performance Liquid Chromatography (HPLC; Agilent Infinity 1260 II with binary pump, refrigerated autosampler, integrated column compartment and variable wavelength detector; Agilent Technologies, Santa Clara, CA, USA). A Reprospher column 100 C18, 5 μm; 12.5 cm x 4.6 mm (Dr. Maisch GmbH, Ammerbuch, Germany) was used with a column temperature of 21°C. A linear gradient of mobile phase A (0.1 M aqueous NaH_2_PO_4_ buffer) and mobile phase B (acetonitrile/water 1:1) was used: 0 min, 30% B; 10 min: 40% B; 17.5 min: 65% B; 20.5 min, 65% B; 23.5 min, 30% B; 26 min, 30% B. Flow rate: 1.5 ml/min. Detection: 220 nm. Injection volume: 70 μl. The diluent used to prepare standard solutions was prepared by dissolving 500 mg of chlorobutanol in 0.5 ml glacial acetic acid, adding 500 mg of ethyl alcohol, 110 mg of sodium acetate and filling up to 100 ml with bi-distilled water. Analysis was performed for three different vials per sample, each injected twice, yielding six measurements per sample. Oxytocin USP Reference Standard purchased from Merck KGaA, Darmstadt, Germany, was used for comparison. The pH value was measured twice for each vial, testing three vials from each sample of oxytocin, and the average value was calculated.

The concentration of the preservative benzyl alcohol, which was only contained in the oxytocin samples manufactured by Jiangxi Xierkangtai Pharmaceutical Co. Ltd, China, was determined using a method modified from Rego and Nelson [33]. The analysis was carried out using HPLC (Agilent 1200 Series with a diode array detector; Agilent Technologies, Santa Clara, CA, USA), with the same column as described above. The mobile phase was composed of 20% acetonitrile and 80% water. Flow rate: 1.5 mL/min. Detection: 254 nm. Injection volume: 50 μl. Samples were diluted 1:30 with bi-distilled water prior to injection, except for sample QOR04 which was injected without dilution. Analysis was performed for at least two different vials per sample, each injected twice. Benzyl alcohol pharmaceutical secondary standard (SIGMA-ALDRICH, St. Louis, MO, USA; Lot LRAC1678; certified purity of 99.98%) was used as reference material.

Misoprostol tablets were analyzed following the respective monograph of the International Pharmacopoeia 2017 (Ph. Int. 2017) for identity, assay and dissolution testing. The Agilent Infinity 1260 II HPLC instrument described above was used with a stainless-steel column packed with ReproSil-XR 120 C18, 5 μm, 150 mm x 4.6 mm (Dr. Maisch GmbH, Ammerbuch, Germany) and a guard column containing the same material. The column oven was kept at 35.0°C. A premixed mobile phase composed of acetonitrile and bi-distilled water in a ratio of 45:55 was used, at a flow rate of 1.5 ml/min in isocratic mode. The injection volume was 100 μl for assay and 250 μl for dissolution testing. HPLC vials were kept in a cooled autosampler at 4°C until sample injection to avoid degradation. Detection was carried out by UV at 200 nm. Sample and standard solutions were freshly prepared using the mobile phase as diluent. For misoprostol assay, two separate determinations were carried out for each sample. For each determination, five tablets were placed into 50 ml of the mobile phase. In the case of three of the collected samples, the number of available tablets was insufficient for this procedure, and in these cases for each of the two determinations one tablet was placed into 10 ml of the mobile phase. Misoprostol was dissolved from the tablets using an ultrasonic bath, and ice was added to the bath to avoid degradation by heat. The solution was filtered through Rotilabo PTFE 0.20 μm filters (Carl Roth GmbH & Co. KG, Karlsruhe, Germany) and injected into the HPLC, with three injections of each of the two solutions, yielding six measurements per sample. European Pharmacopoeia Reference Standard (batch N° 3.0) from the European Directorate for the Quality of Medicines (EDQM) was used for comparison.

Misoprostol tablets were analyzed for related substances according to Ph. Int., using the same HPLC instrumentation and the same column as described above for the assay. The mobile phases consisted of acetonitrile, water and methanol in the ratio 28:69:3 (mobile phase A) and 47:50:3 (mobile phase B), and were used in the gradient mode described in Ph. Int., at a flow rate of 1.5 ml/min and a column temperature of 35°C. The injection volume was 200 μl. Detection was carried out by UV at 200 nm. Sample solutions were prepared as described in Ph. Int. For comparison, a misoprostol standard solution 20 μg/ml in mobile phase A, and the same standard solution heated for 1 hour at 80°C, were used. Each of the sample and standard solutions was injected twice. The different degradation products of misoprostol (Fig 1) were identified as described by Ph.Int., based on their relative retention times compared to misoprostol.

Dissolution testing was conducted according to Ph. Int. using a dissolution tester PT-WS 610 (Pharma Test Apparatebau AG, Hainburg, Germany). Into each of the six dissolution vessels filled with 500 ml of de-ionized water, one tablet was placed. The test was conducted at 37 ± 0.5°C with a paddle rotational speed of 50 revolutions per minute. Samples were withdrawn after 30 min through an in-line filter, and 250 μl of these solutions were injected into the Infinity 1260 II HPLC system described above.

The system suitability for the method for oxytocin assay was verified according to USP 40, and for the methods for misoprostol assay and dissolution according to Ph. Int.

For the determination of the benzyl alcohol concentration, linearity and precision of the applied method were validated according to the International Council for Harmonization (ICH) guideline Q2(R1) [34]. Relative standard deviation of the measurements (repeatability) was 0.2%.

Sample analysis was conducted unblinded to packaging. All oxytocin and misoprostol samples were analyzed before reaching their expiry dates.

### Mass spectrometric analysis

Gas chromatography-mass spectrometry (GC-MS) was carried out using a Hewlett Packard/Agilent HP6890 GC system coupled with a HP5973 mass selective detector. The injector temperature was 280°C. An Agilent HP-5ms Ultra Inert (5%-phenyl)-methylpolysiloxane column 30 m x 0.25 mm with a film thickness 0.25 μm was used. The temperature gradient was 40 to 320°C with 10°C/min, followed by 10 min 320°C isothermal. Helium was used as carrier gas with a flow rate of 1.2 ml/min. Electron impact ionization (EI) was carried out with 70 eV, and a single quadrupole analyzer was used.

HPLC-MS/MS analysis was conducted on a Thermo Scientific UltiMate 3000 HPLC-System coupled with an ESI-TOF Bruker maXis 4G (Bruker Daltonics, Bremen, Germany) in the positive mode.

### Definitions for substandard and falsified medicines

Samples were classified as within specification or out of specification (= substandard) based on the criteria of USP 40 for assay and pH value in case of oxytocin injections, and based on the criteria of the Ph. Int. 2017 for assay and dissolution in case of misoprostol tablets. According to these pharmacopoeias, both oxytocin injections and misoprostol tablets must contain not less than 90.0% and not more than 110.0% of the declared amount of the active pharmaceutical ingredient (API). For oxytocin vials, the pH value must be between 3.0 and 5.0. For dissolution testing of misoprostol tablets, the amount in solution must not be less than 80% (Q) of the amount declared on the label.

Following the terminology introduced by earlier studies of WHO, assay results deviating more than 20% from the declared API content, and dissolution results falling more than 25% below the pharmacopeial Q value, were considered as extreme deviations [35, 36]. Lesser deviations from pharmacopeial specifications were considered as moderate deviations. As per definition of WHO [11], products that deliberately/fraudulently misrepresent their identity, composition or source were considered falsified.

### Data analysis

Excel (Microsoft Office Professional Plus 2019) was used to calculate means, medians and percentiles, and relative standard deviations (RSD). Figures of the distribution of the assay test results for oxytocin injections and misoprostol tablets, and the dissolution test results for misoprostol tablets, were generated using the statistical software JMP 14.2 (SAS GmbH, Heidelberg, Germany).

### Information of national authorities and stakeholders

Following the request stated in the permission No 20/1361/DGPHFIS/2018 from the Ministry of Health of Rwanda to conduct the present study, the authors have submitted on December 2, 2018, an alert letter to the Rwandan authorities about two extremely substandard brands of misoprostol tablets found to circulate in Rwanda (see Results section). Furthermore, this manuscript was shared with the Rwandan Food and Drug Authority (RFDA) and with the WHO Rapid Alert System.

## Results

### Overview of sampling sites

As shown in Table 1, oxytocics could be collected from 40 of the 57 randomly selected health facilities and retail pharmacies, as well as from three government medical stores and three private wholesalers (see Methods section). In health facilities, samples were collected both from medicine storage rooms and from maternity wards if available.

**Table 1:** Overview of sampling sites for oxytocin injections and misoprostol tablets.

Category of health facility	Number of facilities	Sites in facility	Number of sites	Number of oxytocin samples collected	Number of misoprostol samples collected
Government hospitals	3	storage rooms	3	3	4*
		maternity wards	2	2	1
Faith-based hospitals	5	storage rooms	5	5	5
		maternity wards	5	5	2
Government health centers	12	storage rooms	11	11	2
		maternity wards	6	6	0
Faith-based health centers	12	storage rooms	11	11	1
		maternity wards	7	7	0
Private clinics	1	storage rooms	1	1	0
Retail pharmacies	7	storage rooms	7	0	7
Government central medical store	1	storage rooms	1	2*	0
Government district pharmacies	2	storage rooms	2	1	2
Private wholesalers	3	storage rooms	3	3	1
**Total**	**46**		**64**	**57**	**25**

* Two brands collected in one sampling site.

Notably, oxytocin injections were available in every visited government and faith-based health facility, i.e. both in hospitals and health centers, consistent with the recommendations of the Rwanda National List of Essential Medicines (REML) [9]. According to the REML, misoprostol tablets are expected to be available as oxytocics in hospitals but not in health centers. Indeed, all eight hospitals but only three of the 24 visited health centers, had misoprostol tablets available in a sufficient amount for sampling.

A total of 12 retail pharmacies had been randomly selected and visited, but only seven had misoprostol tablets available, and none stored oxytocin injections. Of the 12 randomly selected private clinics, most stated that they do not offer maternity services, and oxytocin could be collected only from a single private clinic.

A detailed list of all sampling sites, with the numbers of oxytocin vials and misoprostol tablets collected at each site, is shown in S1 Table.

### Overview of collected oxytocin samples

A total of 57 oxytocin samples were collected. All of these were packaged in vials of 1 ml, with a concentration of 10 IU/ml. As shown in Table 2, the 57 samples represented only four different brands and a total of seven different batches. In addition to the facilities listed in Table 1, three further private pharmaceutical wholesalers in Kigali were contacted, as well as the procurement organization for faith-based health facilities, i.e. the Bureau de Formations Médicales Agréés du Rwanda (BUFMAR), but none of these had any other oxytocin brands or batches in stock than those shown in Table 2. Therefore, the preparations listed in Table 2 appear to represent most (or all) oxytocin batches which were in circulation in Rwanda at the time of sample collection.

**Table 2:** List of oxytocin brands and batches collected in this study.

Brand name and stated manufacturer	WHO Pre-Qualified/	Stated storage temperature requirement	Batch N°	Manufacture/	Number of samples
	SRA			Expiry Date	
Oxytocin injection	-	room temperature ^**a**^	1606573	Jun 16 / Jun 19	24
Jiangxi Xierkangtai Pharmaceutical Co. Ltd					
			1604521	Apr 16 / Apr 19	9
China					
Steroxine 10 IU/1 ml ^**b**^	SRA	2–8°C	160042	Feb 16 / Jan 19	1
Laboratoires Sterop					
Belgium			160269	Sep 16 / Aug 19	1
Oxytocin 10 IU/ml	WHO-PQ	2–8°C	37611016	Oct 16 / Oct 20	4
AS Grindeks					
Latvia ^**c**^			37711116	Nov 16 / Nov 20	1
Oxytocin 10	SRA	2–8°C	70779A	Sep 17 / Sep 20	17
Rotexmedica GmbH Arzneimittelwerk					
Germany					

WHO-PQ = WHO-prequalified medicine; SRA = manufactured in a country with stringent regulatory authority.

^a^ Storage requirement stated on packaging: "Store in a cool dry place, away from light"; storage requirement stated on the package insert: "Store in a dark place at room temperature, protect from light."

^b^ Batch 160269 was labeled with the unbranded generic name “Oxytocin 10 IU/1 ml”, all other information was identical as in batch 160042.

^c^ Marketing authorization holder: Peckforton Pharmaceuticals Ltd., United Kingdom.

The most frequently encountered preparation, representing 33 samples, was stated to be manufactured by Jiangxi Xierkangtai Pharmaceutical Co. Ltd, China. On its secondary packaging, the storage requirement was stated as "Store in a cool dry place, away from light", while in the package insert, the storage requirement showed a slightly different wording: "Store in a dark place at room temperature, protect from light."

The three other brands (Table 2) were stated to be manufactured in European countries with stringent regulatory authorities (SRAs) [37], and all of them were labeled for refrigerated storage (i.e. storage at 2–8°C). The product manufactured by AS Grindeks, Latvia (marketing authorization holder: Peckforton Pharmaceuticals Ltd., UK) was a WHO-prequalified medicine [38, 39].

The stated shelf life of three out of the four brands was three years, and four years in case of the preparation by Grindeks. The package inserts of the preparations by Sterop (Belgium), Grindeks (Latvia) and Rotexmedica GmbH Arzneimittelwerk (Germany) stated the excipients, i.e. sodium chloride, different buffering agents, water for injection, and in case of the Sterop preparation also chlorobutanol, a preservative and stabilizing agent [40]. In contrast, no excipients were stated for the product by Jiangxi Xierkangtai.

The preparations by Jiangxi Xierkangtai (China), by Rotexmedica (Germany) and by Grindeks (Latvia) were all found in both government and faith-based facilities. In contrast, the preparation by Sterop (Belgium) was only found in two private pharmaceutical wholesalers at the time of sample collection. No expired samples of oxytocin were found to be in circulation.

### Oxytocin storage conditions

Out of 57 samples of oxytocin, 33 were labeled for room temperature storage, and 24 for refrigerated storage. The actual storage place in the facilities could be inspected at 52 of the 56 sampling sites; it could not be inspected at the pharmaceutical wholesalers which were contacted by a mystery shopper approach. Notably, in all inspected sites the actual storage place (i.e. inside or outside the refrigerator) correctly corresponded to the storage recommendation of the manufacturer.

Temperature data loggers were placed at the storage places of oxytocin and recollected after six months. In five cases, the loggers failed to record data or got lost in the facility, but from 47 oxytocin storage sites temperature recordings were obtained. These comprised 18 refrigerated and 29 non-refrigerated storage places. The results recorded at the individual sites are listed in S1 Table.

In 13 out of the 18 refrigerated oxytocin storage sites, the recorded mean kinetic temperature (MKT) ranged from 4.3°C to 7.3°C, compliant with the manufacturers’ storage requirement of 2–8°C. In one site, the recorded storage temperature was too low (MKT = -1.3°C). At this site, except for short temperature spikes (possibly due to opening of the refrigerator), the recorded temperature was constantly around -2°C. Though this indicates an incorrect temperature setting of the refrigerator, it is unlikely to have caused freezing of the preparation, due to the presence of excipients in the oxytocin vials. In three sites, the recorded MKTs in the refrigerators were slightly too high (8.5°C, 9.1°C and 10.5°C, respectively). And in one further site (a maternity ward of a faith-based hospital), oxytocin was not stored in a refrigerator but in a cool box, reportedly only for immediate use and for not more than 24 hours. Indeed, the temperature data logger at that site recorded alternating periods of cold temperature and room temperature. The MKT in this cool box over the entire recording period resulted as 15.8°C, but the storage temperature at the times of oxytocin storage may well have been correct.

In most of the other storage sites, the temperatures were largely constant over the entire recording period. Only in one case larger fluctuations (between +15°C and -2°C) of longer duration were observed. Five sites showed few very brief periods of temperatures above 8°C which may have been due to occasional power failures, as also mentioned by the staff of these facilities.

At the 29 non-refrigerated oxytocin storage sites, the median value of the recorded MKTs was 23.5°C (range 19.8–26.3°C), reflecting the temperate climate of Rwanda, most of which is situated at an altitude of approximately 1500 m.

### Packaging examination and visual inspection of oxytocin samples

Packaging examination revealed no irregularities except for a few minor mistakes in the use of upper and lower case letters on the packaging and in the package inserts of the product stated to be manufactured by Jiangxi Xierkangtai (China). However, one sampling site (private clinic) stored the vials of oxytocin not in their original secondary packaging but in a box labeled as dexamethasone. Visual inspection showed no deficiencies like color changes or suspended particles.

### Chemical analysis of oxytocin samples

**Fig 3:**
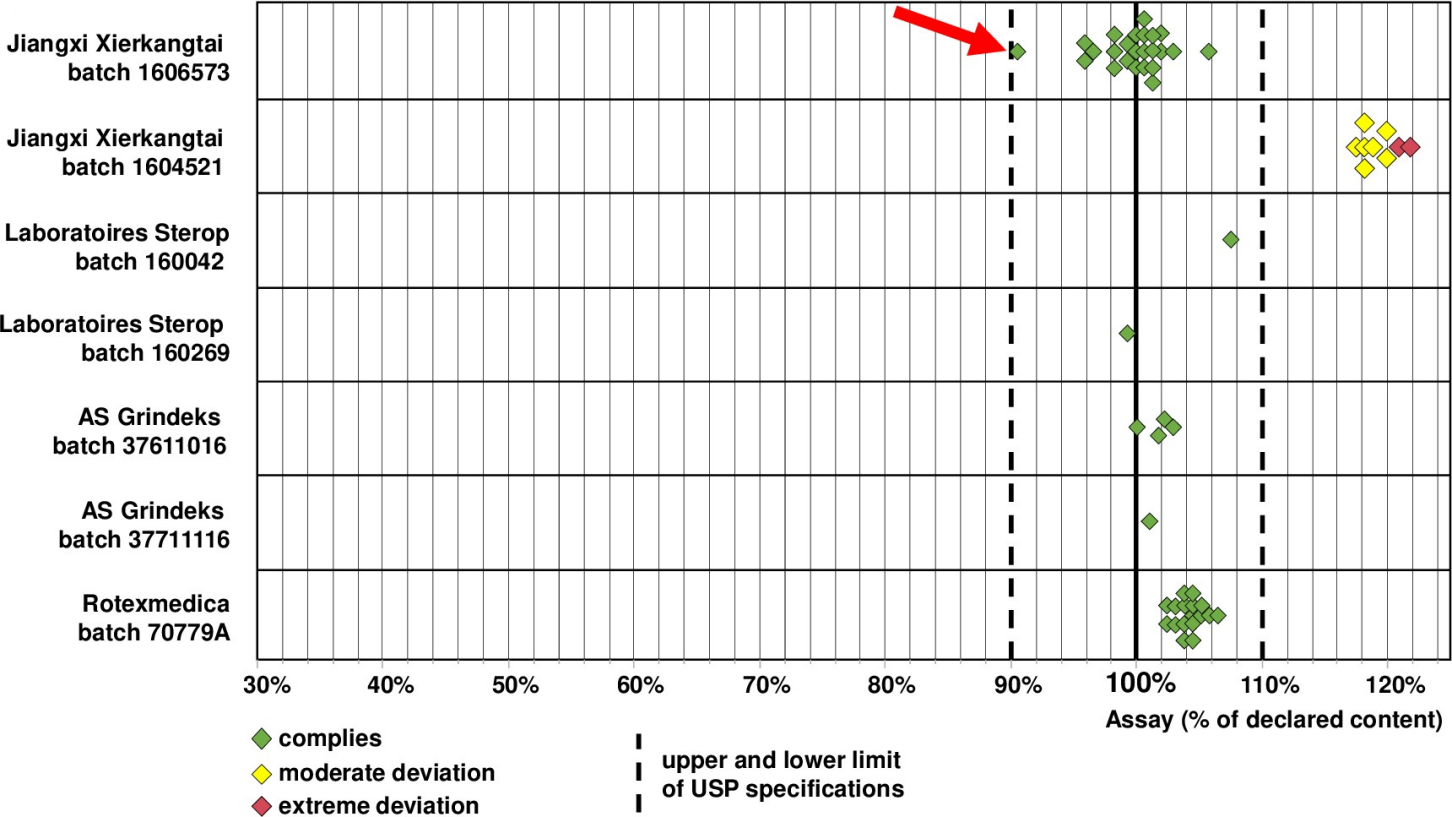
Content of oxytocin determined in each of the investigated samples. The arrow marks a sample with deviating content of the active pharmaceutical ingredient oxytocin and of the preservative benzyl alcohol (see main text and Fig 4).

The presence of oxytocin could be confirmed for all samples, therefore neither packaging examination nor chemical analysis indicated the presence of any falsified oxytocin samples. Also the pH value which is important for oxytocin stability was within USP specifications (3.0–5.0) for all samples (S2 Table). Fig 3 shows the distribution of the assay results for the investigated brands and batches. Notably, none of the 57 samples showed an insufficient content of oxytocin.

The 17 samples of Rotexmedica (Germany) all belonged to one single batch and showed a median content of 103.8% of the declared amount. These samples had been collected at different points of the supply chain (government central medical store; hospitals; government and faith-based health centers), but nevertheless showed a very uniform API content (range 102.0–105.9% of the declared amount). Likewise, the samples of AS Grindeks (Latvia) showed a very uniform content (range 99.9–102.8% of the declared amount). The two samples of Sterop (Belgium), belonging to different batches, contained 99.6 and 107.8% of the declared amount.

In sharp contrast, all nine samples of batch no. 1604521, stated to be manufactured by Jiangxi Xierkangtai (China) were found to deviate from USP specifications (90–110% of the declared amount) by containing too high amounts of oxytocin (median 118.0%). For two of these samples, the obtained assay result even exceeded the declared content by more than 20%, which represents an extreme deviation following the definition used in previous studies of WHO [35, 36].

In the other batch by Jiangxi Xierkangtai (batch no. 1606573), 23 of the 24 samples ranged in their content from 95.6 to 105.5% of the declared amount, well inside the content range specified by USP. However, for one sample of this batch, collected from a private wholesaler (sample no. QOR04; collected from facility no. 44 in S2 Table), the oxytocin content determined for three investigated vials was 89.7%, 90.4% and 91.0% of the declared amount, respectively, therefore on the borderline of USP specifications.

Fig 3 shows that the oxytocin content varied between different brands and batches. The results of the chemical analysis of each oxytocin sample, and the age of the samples at the time of analysis, are shown in S2 Table. In case of Jiangxi Xierkangtai batch no. 1606573, samples which were collected during the pilot study in March 2018 were 24 months old at the time of analysis. These did not show a higher content than those samples which were collected in the main study in September and October 2018 and were 30 months old at time of analysis. This, and the other data in in S2 Table, provide no evidence that differences in the age of the samples were important for the observed differences in oxytocin content.

**Fig 4:**
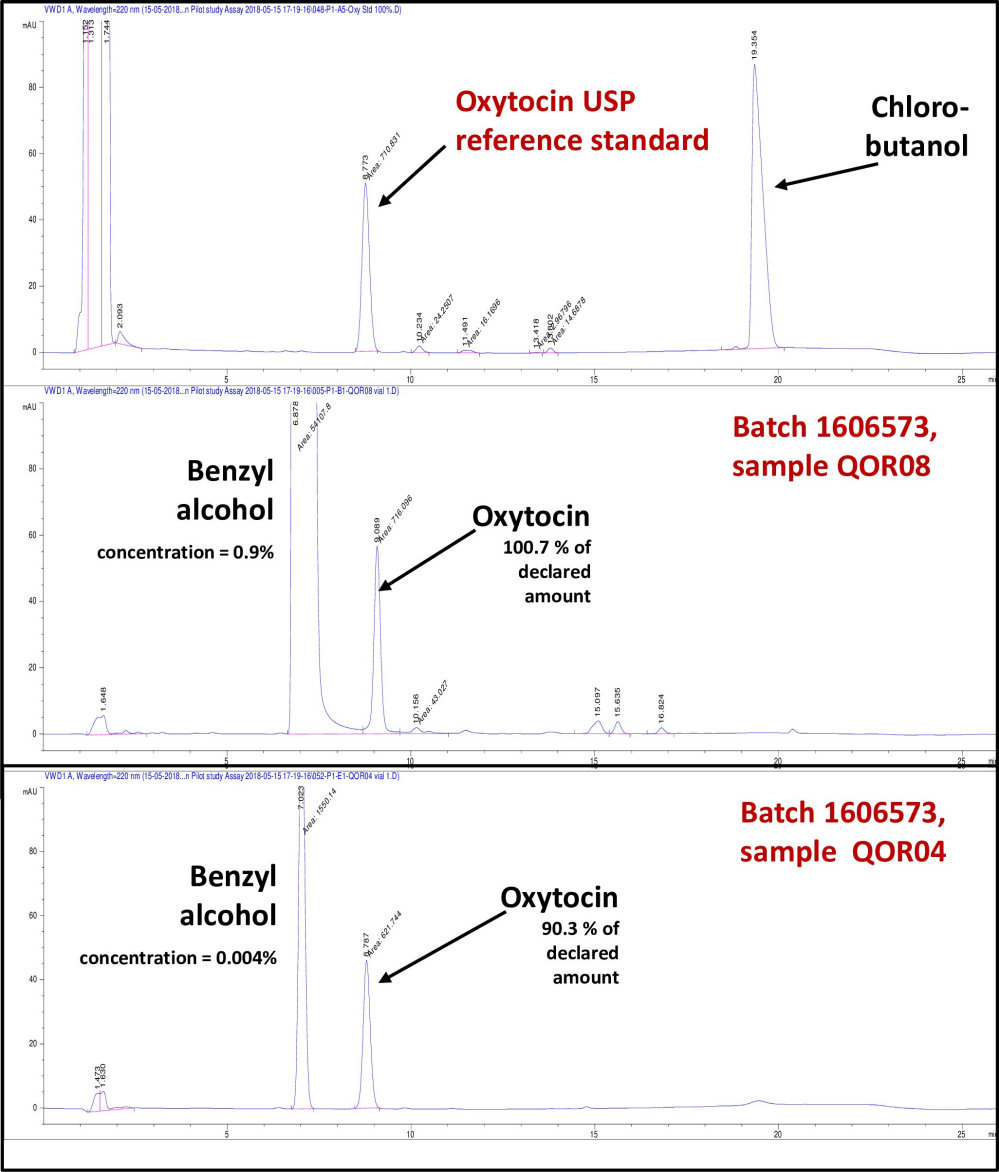
HPLC detection of the undeclared preservative benzyl alcohol, present in different concentrations in oxytocin samples of the same batch, stated to be manufactured by Jiangxi Xierkangtai Pharmaceuticals Co. Ltd (China).

Unexpectedly, the HPLC assay of the samples stating Jiangxi Xierkangtai as manufacturer showed a very large peak of an unknown substance eluting approximately two minutes earlier than oxytocin (Fig 4). Neither the packaging nor the package insert gave any information on the identity of this substance. An enquiry was sent to the three e-mail addresses given on the website of the stated manufacturer, but remained unanswered. Therefore, the identity of the unknown substance was investigated by mass spectrometry. GC-MS analysis showed the following mass and fragmentation: m/z 108 (M^+^; 92%), 107 (67%), 91 (17%), 79 (100%), 77 (63%), 65 (7%), 51 (12%), 39 (8%). Comparison to the database of the National Institute of Standards and Technologies, Gaithersburg, MD, USA (NIST; http://webbook.nist.gov) showed that these data were identical to those of benzyl alcohol, a commonly used preservative in parenteral pharmaceutical preparations [41]. Subsequently, the identity of the unknown substance was confirmed by both GC-MS and HPLC-MS investigation in comparison to authentic benzyl alcohol, showing identical retention times and mass spectrometric fragmentations. The concentration of benzyl alcohol was determined as 0.9% in nearly all samples.

However, the oxytocin sample no. QOR04 (which had already been noticed to contain the lowest oxytocin amount of all investigated samples, see above) was found to contain only 0.004% benzyl alcohol, showing identical concentrations in all vials. This sample had been collected from a private wholesaler in Kigali. It is highlighted in Fig 3 by an arrow, and in S2 Table by bold print. Its batch number as well as primary and secondary packaging and package insert were identical to those from the 23 other samples which stated Jiangxi Xierkangtai as manufacturer (S2 Fig).

Within all other investigated samples by Jiangxi Xierkangtai only a single vial was found with a deviating concentration of benzyl alcohol. It belonged to a sample (no. QOR75) which had been collected in a government district hospital (listed as facility no. 2 in S1 Table). That vial showed a concentration of 0.018% benzyl alcohol and an oxytocin content of 88.3% of the declared amount. The other two investigated vials of the same sample QOR075 carried the same batch number and had the same appearance, but showed a concentration of 0.9% benzyl alcohol and oxytocin contents of 99.7 and 100.2% of the declared amount, respectively.

### Overview of collected misoprostol samples

Twenty-five samples of misoprostol tablets were collected. All of them had a stated content of 200 μg per tablet. These samples represented six brands and a total of ten batches (Table 3). As mentioned above for oxytocin, also three further wholesalers as well as the faith-based procurement agency BUFMAR were contacted, but had no additional brands or batches of misoprostol tablets in stock. The preparations listed in Table 3 therefore appear to represent most (or all) of the batches of misoprostol tablets which were in circulation in Rwanda at the time of sample collection.

**Table 3:** List of misoprostol brands and batches collected in this study.

Brand name and stated manufacturer	WHO Pre-qualified/	Stated storage requirements	Batch N°	Manufacture/	Number of samples
	SRA			Expiry Date	
	SRA	No special storage requirements ^**c**^	B15445 ^**a**^	Dec 16 ^**d**^ / Nov 19	2
Cytotec^®^ 200 μg			B17173 ^**a**^	Jul 17 ^**d**^ / Jun 20	6
Piramal Healthcare UK Limited		Store at room temperature (15–25°C)	B18097 ^**b**^	Nov 17 ^**d**^ / Oct 20	2
United Kingdom					

Ace Miso^®^	WHO-PQ	Do not store above 30°C, protect from light	ACE160963	Sep 16 / Aug 18	1
Acme Formulation Pvt. Ltd.					
India					
MIZO^®^	-	Store at a temperature not exceeding 30°C at a dry place	E6SGFT010	Jun 16 / May 18	1
SYNOKEM Pharmaceuticals LTD India			E6SGLT004	Dec 16 / Nov 18	1
Misoprostol 200 mcg Tablets	WHO-PQ	Store at a temperature not exceeding 30°C	45180301	Feb 18 / Feb 20	2
China Resources,					
ZIZHU Pharmaceuticals Co Ltd					
China					
C-stol^®^	-	Store below 30°C. Protect from light and moisture	ERW-005	Mar 18 / Feb 21	3
CORONA Remedies Pvt Ltd					
India					
Cynomax^®^	-	Store at 20 to 25°C in a dry area	M8TAB1801	May 18/ Apr 20	4
MAXTAR BIO-GENICS					
India			MTYX-1604	Aug 16 / Jul 18	3

WHO-PQ = WHO-prequalified medicine; SRA = manufactured in a country with stringent regulatory authority.

^a^ Marketing authorization holder: Pfizer Holding, France.

^b^ Marketing authorization holder: Continental Pharma Inc., Belgium.

^c^ Package insert: "Tenir hors de la vue et de la portée des enfants. Pas de précaution particulière de conservation". I.e.: "Keep out of sight and reach of children. No special storage requirements."

^d^ Manufacturing date not stated on packaging. Shelf-life listed according to information from the websites www.hpra.ie and www.medicines.org.uk/emc.

The most frequently encountered preparation, representing ten of the 25 collected samples, was the originator product Cytotec^®^. For two of the three collected batches, the marketing authorization holder was Pfizer Holding (France), and for the third batch it was Continental Pharma Inc., Belgium (see Table 3). For all three batches, the stated manufacturer of the collected samples was Piramal Healthcare, based in the United Kingdom and therefore in a country with a stringent regulatory authority (SRA). Three further samples were WHO-prequalified medicines [38, 39] and had been produced by Acme Formulation Pvt Ltd, India, or by China Resources Zizhu Pharmaceutical Co, Ltd, China, respectively. The remaining 12 samples represented three generic preparations which were manufactured in India (Table 3), a country without an SRA, and were not WHO-prequalified.

Storage requirements were stated on the packaging and/or in the packaging insert of most misoprostol samples, but the indicated temperatures varied (Table 3): two samples were labeled for storage at 15–25°C, seven samples at 20–25°C, and eight samples at ≤ 30°C. For the remaining eight samples, representing two of the three batches of the originator product Cytotec^®^, the package insert surprisingly stated: “No special storage requirements.”

Misoprostol is very unstable and must be formulated as a 1% dispersion in hydroxypropyl methylcellulose (HPMC; hypromellose) to protect it from degradation [29]. For both the originator product and the WHO-prequalified product by Acme (India), exactly this formulation was stated in the package insert. Zizhu Pharmaceuticals, Co (China), listed hypromellose as an excipient for its product. The Indian company Synokem Pharmaceuticals Ltd (Mizo^®^) mentioned that misoprostol was contained as a “1% dispersion”, but failed to mention what it was dispersed in. And notably, for the products by the Indian companies Corona Remedies Pvt., Ltd and Maxtar Bio-Genics, no information on excipients was given, and it remained unclear whether or not HPMC was contained therein.

The shelf life of four of the brands was given as two years, while for C-stol^®^ (Corona Remedies, India) the stated shelf life was three years. For the originator medicine Cytotec^®^ (Piramal Health Care, UK), only an expiry date but no manufacturing date was given on the packaging, but internet databases stated a shelf life of three years for this brand (see Table 3).

### Misoprostol blister materials and storage conditions

While oxytocin degradation is primarily caused by elevated storage temperatures, misoprostol degradation is especially caused by exposure to moisture [27, 28]. Therefore, misoprostol tablets must be packaged in double-sided aluminium blisters, not in conventional plastic-aluminium blisters. Indeed, all collected samples were correctly packaged in double-sided aluminium blisters.

In the present study, storage temperatures were systematically recorded for oxytocin but not for misoprostol. However, the storage temperatures recorded at the 29 non-refrigerated oxytocin storage sites, as well as in four of the investigated retail pharmacies (S1 Table) may present a good approximation for the misoprostol storage temperatures in health facilities and drug outlets of Rwanda. As mentioned above, the recorded mean kinetic temperatures (MKTs) were moderate, and only in five out of 33 sites they slightly exceeded 25°C (with recorded MKTs between 25.1 and 26.3°C). From one of these sites (MKT 25.4°C), a misoprostol sample with a stated storage requirement of 20–25°C was collected (S1 Table), i.e. the MKT exceeded the demanded storage temperature by 0.4°C. Therefore, in the present study no serious violations of the recommendations for packaging and for storage temperatures of misoprostol tablets were observed.

### Chemical analysis of misoprostol samples

**Fig 5:**
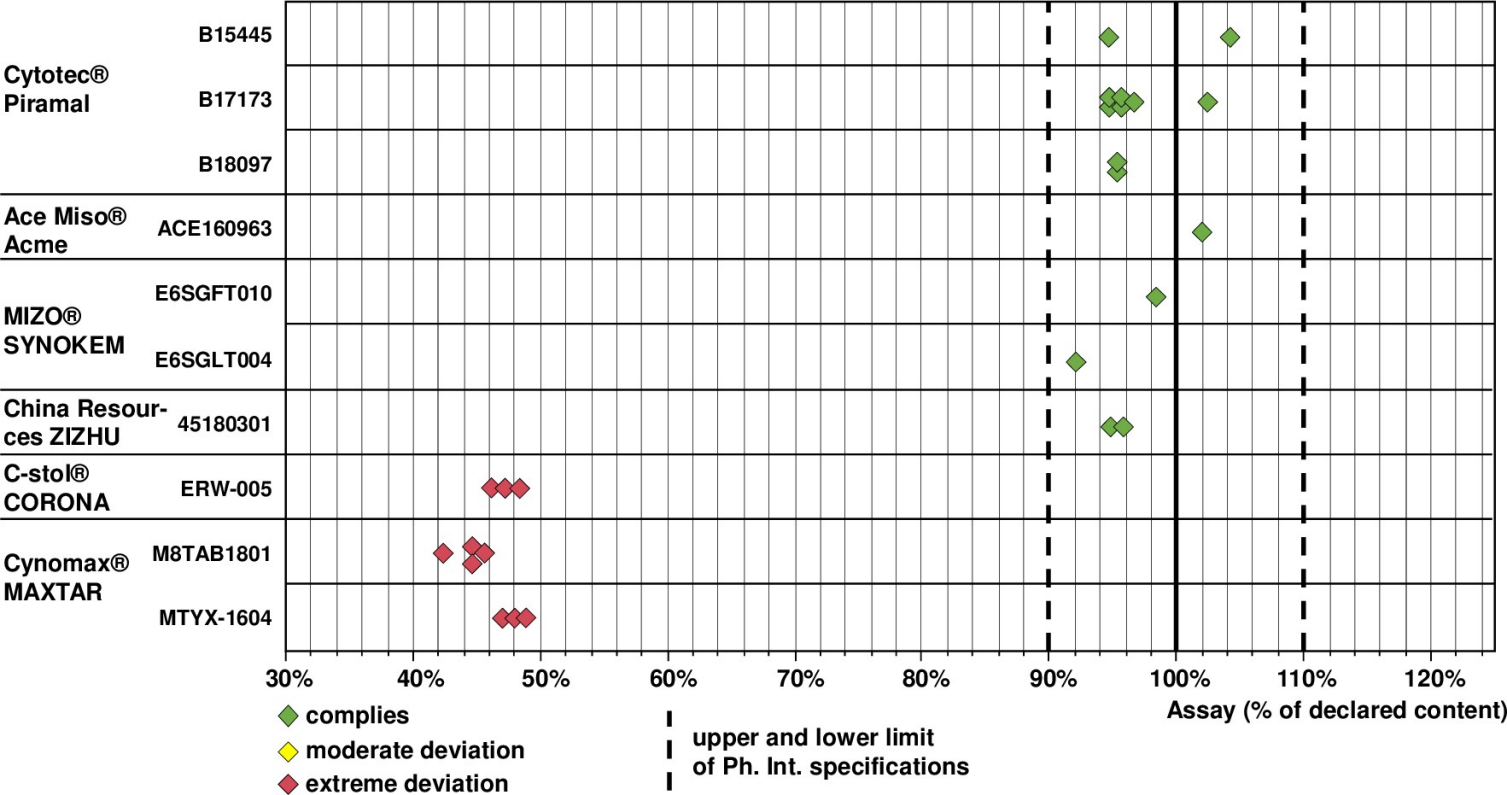
Content of misoprostol determined in each of the investigated samples.

The presence of misoprostol was confirmed in all samples, but assay and dissolution testing revealed dramatic shortcomings in two of the six investigated brands. As shown in Fig 5, the assay results were within Ph. Int. specification (90–110% of the declared amount) for all samples of the originator product, of the two WHO-prequalified brands and of the product by Synokem Pharmaceuticals, India. In sharp contrast, all three samples of C-stol^®^ and all seven samples of Cynomax^®^ showed less than 50% of the declared content, i.e. failed assay testing with extreme deviations. Therefore, an additional HPLC analysis was carried out for these samples, using the method of Ph. Int. for detection of related substances. This clearly showed large amounts of the typical degradation products of misoprostol (S3 Fig), suggesting that the low content of misoprostol in these two preparations was due to degradation of the API. The two investigated batches of Cynomax^®^ had different ages at the time of analysis (7 and 22 months since the date of manufacture; S3 Table) and showed different API contents (mean 44.4% and 47.8% of the declared content). However, contrary to expectations the higher content was recorded for the older sample, indicating that the difference in content may not have been due to different age but due to differences in manufacture; different transport and storage conditions represent another possible reason.

**Fig 6:**
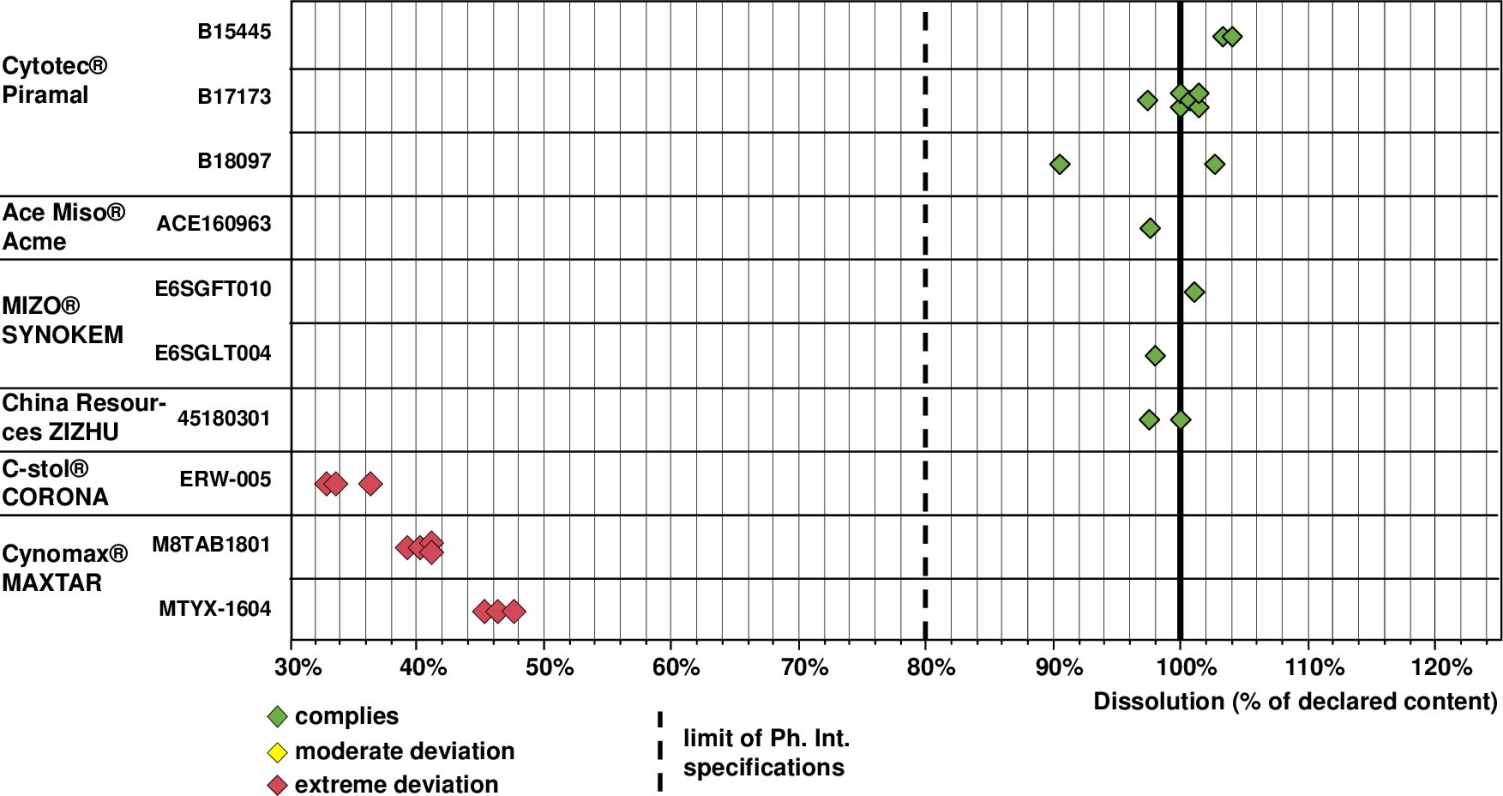
Dissolution of misoprostol determined in each of the investigated samples.

All misoprostol samples were also tested for dissolution of the API. The International Pharmacopoeia demands that from misoprostol tablets at least 80% of the declared API amount must be released in 30 min under the defined conditions. As shown in Fig 6, all samples which had passed assay testing also passed dissolution testing. However, the samples of C-stol^®^ and Cynomax^®^, which had been shown already in assay testing to contain less than 50% of the declared API amount, obviously failed dissolution testing with extreme deviations from USP specifications. From the C-stol^®^ samples, approximately one quarter of the contained amount of the API did not dissolve, proving shortcomings in dissolution in addition to the extreme non-compliance in the assay. The results of the chemical analysis of each misoprostol sample, and the age of the samples at the time of analysis, are shown in S3 Table.

The two extremely non-compliant brands had been collected both from government and from faith-based health facilities, and from one retail pharmacy.

### Product recall in Rwanda

The Rwanda Food and Drug Authority (RFDA) was alerted by the authors of this study about the two extremely substandard brands of misoprostol tablets by e-mail on December 2, 2018. RFDA invited the authors to a meeting at RFDA which took place on December 4, 2018. On December 7, 2018 RFDA issued an alert (Ref 079/Rwanda FDA/2018) requesting all suppliers and retailers to stop the distribution of these products and to put them in quarantine; all public and private hospitals, health centers, clinics and retail pharmacies were instructed to stop dispensing these products until investigations on the quality issues by Rwanda FDA were completed. Subsequently, RFDA issued a formal recall (Ref 0108/Rwanda FDA/2019 of 13 February 2019), stating that RFDA’s investigations had revealed the indicted batches of misoprostol tablets to be substandard, and instructing all wholesalers, retailers, district pharmacies, public and private health facilities that the indicted batches should be returned to the supplier for suitable disposal.

## Discussion

The present study showed excellent (100%) availability of oxytocin injections in the investigated government and faith-based health facilities. Also misoprostol tablets were available as oxytocics in all investigated hospitals, as foreseen by the Rwanda National List of Essential Medicines [9]. This proves remarkable success of the health authorities of Rwanda in assuring the availability of these life-saving commodities in hospitals and health centers.

Oxytocin storage inside or outside the refrigerator was found to exactly follow the manufacturers’ instructions. For samples labeled for storage at 2–8°C, the correct storage temperature was maintained well in most of the sites, with only few and probably inconsequential deviations. Again, this proves success of the health authorities of Rwanda, and stands in positive contrast to reports on oxytocin storage conditions in many other LMICs [19, 24, 25, 42–45]. A direct consequence of this success may be the fact that not a single sample of oxytocin was found which had an insufficient content of the API, again in contrast to reports from many other countries [22, 24, 25]. Of course, it cannot be excluded that knowledge of being monitored in this study may have influenced the behavior of health facility staff.

The number of different brands of oxytocin and misoprostol circulating in Rwanda at the time of sample collection was remarkably small. This may be related to the small market size of Rwanda: many manufacturers and international distributors may not see sufficient economic incentive to engage in medicine sales in this country. Possibly, this can limit the ability of public and private stakeholders to select good-quality, affordable medicines in their procurement.

Out of ten brands collected in total (4 of oxytocin injections, 6 of misoprostol tablets), three were prequalified by WHO [38, 39], and three more were manufactured in countries with stringent regulatory authorities (SRAs) [37]. Together, these six brands represented 45% of the samples collected in this study, and notably all these samples passed the quality tests in this study with good results, indicating that prequalification by WHO, as well as production in a country with an SRA, is a reliable predictor of good quality of medicines.

In stark contrast, three out of the four brands which were neither WHO-prequalified nor produced in a country with an SRA showed serious quality deficiencies. Most alarmingly, every investigated sample of the misoprostol products Cynomax^®^ (stated manufacturer Maxtar Bio-Genics, India) and C-stol^®^ (stated manufacturer Corona Remedies, India) failed assay and dissolution testing with extreme deviations from the pharmacopeial specifications, likely to result in clinical inefficacy. Large amounts of misoprostol degradation products were detected in these two brands, indicating that the API had degraded to a large extent. Notably, the package inserts of both preparations did not mention whether or not HPMC (hypromellose) had been used in the formulation of these tablets, which is essential for misoprostol stability [29]. Also in Malawi, extremely substandard misoprostol brands, however from different stated manufacturers, have been found [24].

The quality deficiencies observed for the oxytocin injection stating Jiangxi Xierkangtai (China) as manufacturer were not as extreme as those observed for the above-mentioned misoprostol brands, but still alarming. All six samples of one of the two investigated batches of that oxytocin brand exceeded the content limit specified by USP, two of the samples even contained more that 120% of the stated amount. In pharmaceuticals with unstable APIs, a content slightly higher than 100% of the stated amount is often intentionally included to ensure that the content is still above the lower pharmacopeial limit towards the end of the shelf life. However, the pharmacopeias define an upper limit to avoid overdosing of the therapy, and this limit was clearly exceeded in the batch in question.

The oxytocin injections stating Jiangxi Xierkangtai as manufacturer were found to contain benzyl alcohol in a concentration of 0.9%. This compound in this concentration is perfectly acceptable as a preservative in parenteral preparations [39, 46]. However, in most countries regulations demand that the presence of such excipients must be declared in the package insert [47], but for the product in question no excipients were declared at all.

The most worrying observation regarding this product, however, was the detection of vials with a two-hundred-fold lower benzyl alcohol concentration, carrying the same batch number as the vials with 0.9% of that preservative. While this may not cause direct harm to a patient treated with that product, it indicates gross violations of good manufacturing practice, raising strong doubts also about other quality aspects of this product. This kind of problem has not been reported in previous studies of oxytocin quality. However, this problem may easily escape detection in a medicine quality study as it has been visible only in a small number of the investigated vials.

The oxytocin injections by Jiangxi Xierkangtai were labeled for non-refrigerated storage, as also is the case for oxytocin from many other manufacturers [19, 24, 42–44]. Obviously, the use of oxytocin products which do not have to be stored in a refrigerator appears to be an attractive option, especially in LMICs. However, recent studies have shown that oxytocin products labeled for non-refrigerated storage may not have any better stability than products labeled for refrigerated storage, and on the contrary may even be less stable [40, 44]. Therefore, international stakeholders including WHO have issued the recommendation “Buy quality oxytocin, keep it cool” and recommended that all oxytocin products should be stored at 2–8°C [16, 48], irrespective of the manufacturer’s storage recommendation. Health authorities in Rwanda and elsewhere may consider this recommendation. A heat-stable formulation of the oxytocin analogue carbetocin has recently been added to the WHO Essential Medicines List, and in future it may become a further treatment option for post-partum hemorrhage for use in facilities where storage at 2–8°C is problematic.

Several previous studies have reported problems of oxytocin quality in LMICs, but the present study shows for Rwanda a decidedly different situation than reported from other countries. The present data suggest that the Rwandan authorities have successfully assured the availability and (in most cases) the appropriate storage of oxytocin according to manufacturers’ instructions. The detected problems of oxytocin and especially misoprostol quality must be addressed not so much by improved storage and transportation conditions of the medicines, but by improvements of the supplier qualification in medicine procurement. It may be considered whether for oxytocin and misoprostol, with their well-known problems of quality and stability, procurement should be restricted to WHO-prequalified medicines and to medicines manufactured in countries with stringent regulatory authorities. And as a simple “rule of thumb”, the results of this study suggest that medicines for which the excipients are not stated in the package insert should be regarded as doubtful in quality.

So far, misoprostol quality has received much less attention in scientific studies than oxytocin quality [14]. However, the results of this study, of the study of Hall [28] and of a recent study from Malawi [24], show quality problems of different brands of misoprostol which are even much more serious than those reported for oxytocin, with API contents below (or far below) 50% of the stated amount, and this problem needs attention in future studies.

Rwanda has only recently established its national drug regulatory agency, i.e. the Rwanda Food and Drug Authority (RFDA), and this will certainly contribute to increased patient safety. The extremely quick and efficient action which RFDA took on the reported substandard misoprostol brands holds good promise for the future development of medicine quality in this country.

## Supporting information

S1 Fig Sample site & drug purchase record. (PDF)

S2 Fig Photos of two samples of oxytocin injections, carrying the same batch number but containing different concentrations of benzyl alcohol. (PDF)

S3 Fig HPLC analysis for related substances in two misoprostol samples. (PDF)

S1 Table List of included health facilities, number oxytocin vial and misoprostol tablets collected, and recorded oxytocin storage conditions. (PDF)

S2 Table Results of chemical analysis of all oxytocin samples. (PDF)

S3 Table Results of chemical analysis of all misoprostol samples. (PDF)
