# Supplementary material for: Quality of oxytocin and misoprostol in health facilities of Rwanda
Source: PLoS One. 2021 Jan 8;16(1):e0245054. doi: 10.1371/journal.pone.0245054 (PMC7793248; doi:10.1371/journal.pone.0245054)
Supplement: S1 Fig — (PDF) [file pone.0245054.s001.pdf]

SAMPLE SITE & DRUG PURCHASE RECORD

Name of survey site: ____Date and time of visit: ______________________

Description of location: ____

Temperature data logger Location: ____ Serial number: ____

Photo taken (5): YES ☐ NO ☐

Drug samples collected	Desired quantity	Obtained quantity	QOR reference number	Reason for not obtaining the desired quantity	Brand name	Batch no	Expiry date	Manufacturer, country	Price in Rwf per tabs/vial (if applicable)	Storage conditions according to the manufacturer		Refrigerated (r) / not refrigerated (nr) in original package (op)/out of original package (nop) protected from light (pr) /not protected from light (npr)	1) Thermometer kept with oxytocics? 2) Temperature recorded daily? If yes, please take picture
										Temperature in °C	Relative humidity in %		
**Misoprostol 0,2mg tab.**	50											(r) ☐ (nr) ☐<br>(op) ☐ (nop) ☐<br>(pr) ☐ (npr) ☐	1) yes ☐ no ☐<br>2) yes ☐ no ☐
**Oxytocin 10 IU**	10											(r) ☐ (nr) ☐<br>(op) ☐ (nop) ☐<br>(pr) ☐ (npr) ☐	1) yes ☐ no ☐<br>2) yes ☐ no ☐

- **If samples are taken without original package, please take picture of original package (showing the name, batch number, expiry date, manufacturing date, name / address of manufacturer)!**
- Collected samples were replaced: YES ☐ NO ⍰ Collected samples were paid for: YES ⍰ (Attach receipt!) NO ⍰
- Name of sampling person: ____Signature: _________________________
- Name of person responsible for health facility: ____Signature: _________________________

S1 Fig. Sample site & drug purchase record.
