# Supplementary material for: Quality of oxytocin and misoprostol in health facilities of Rwanda
Source: PLoS One. 2021 Jan 8;16(1):e0245054. doi: 10.1371/journal.pone.0245054 (PMC7793248; doi:10.1371/journal.pone.0245054)
Supplement: S2 Fig — (PDF) [file pone.0245054.s002.pdf]

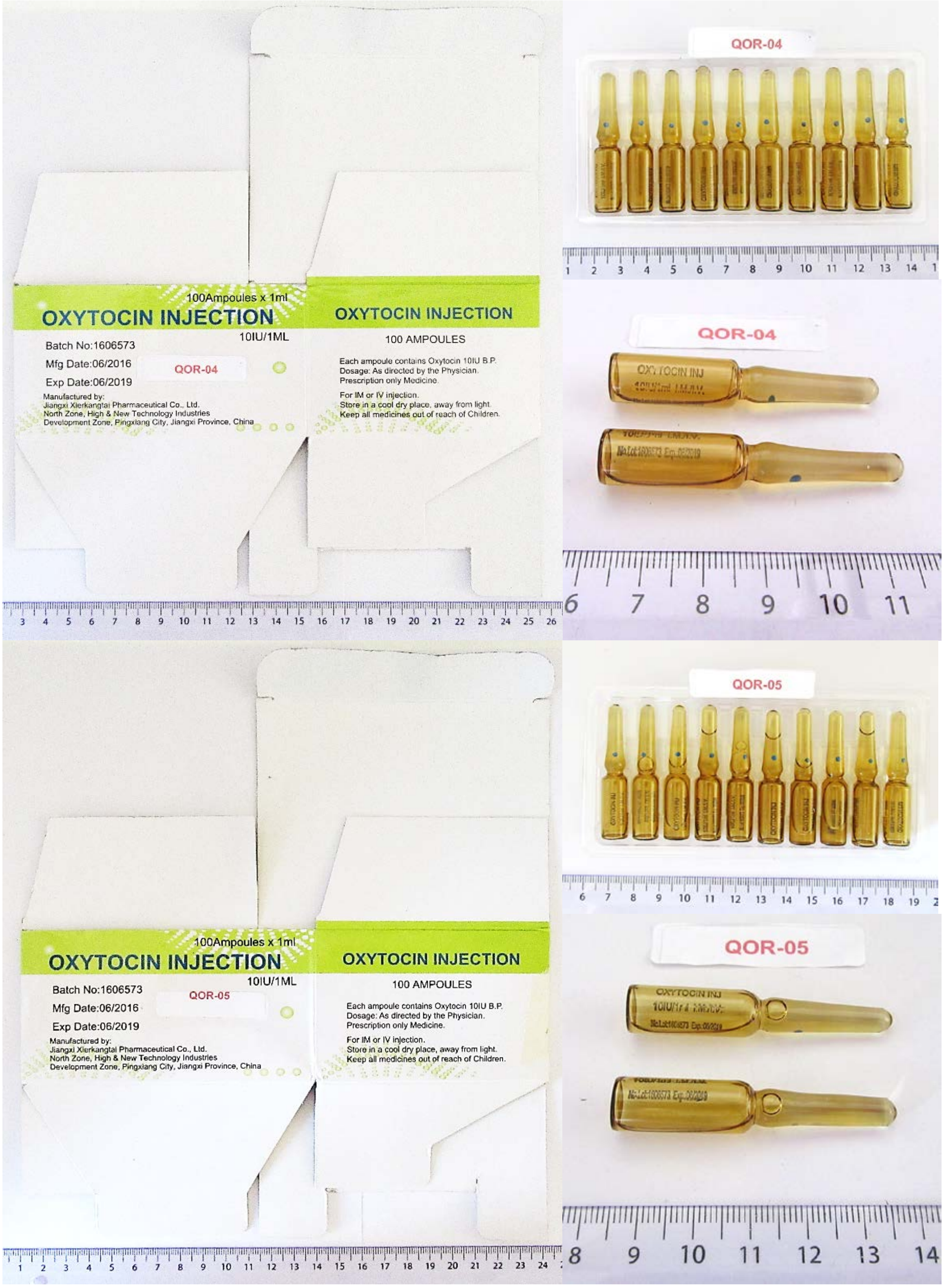

S2 Fig: Photos of two samples of oxytocin injections, carrying the same batch number but containing different concentrations of benzyl alcohol.

Top: Sample No. QOR04, containing 0.004% benzyl alcohol.
Bottom: Sample No. QOR05, containing 0.9% benzyl alcohol.
