# Supplementary material for: Quality of oxytocin and misoprostol in health facilities of Rwanda
Source: PLoS One. 2021 Jan 8;16(1):e0245054. doi: 10.1371/journal.pone.0245054 (PMC7793248; doi:10.1371/journal.pone.0245054)
Supplement: S3 Fig — (PDF) [file pone.0245054.s003.pdf]

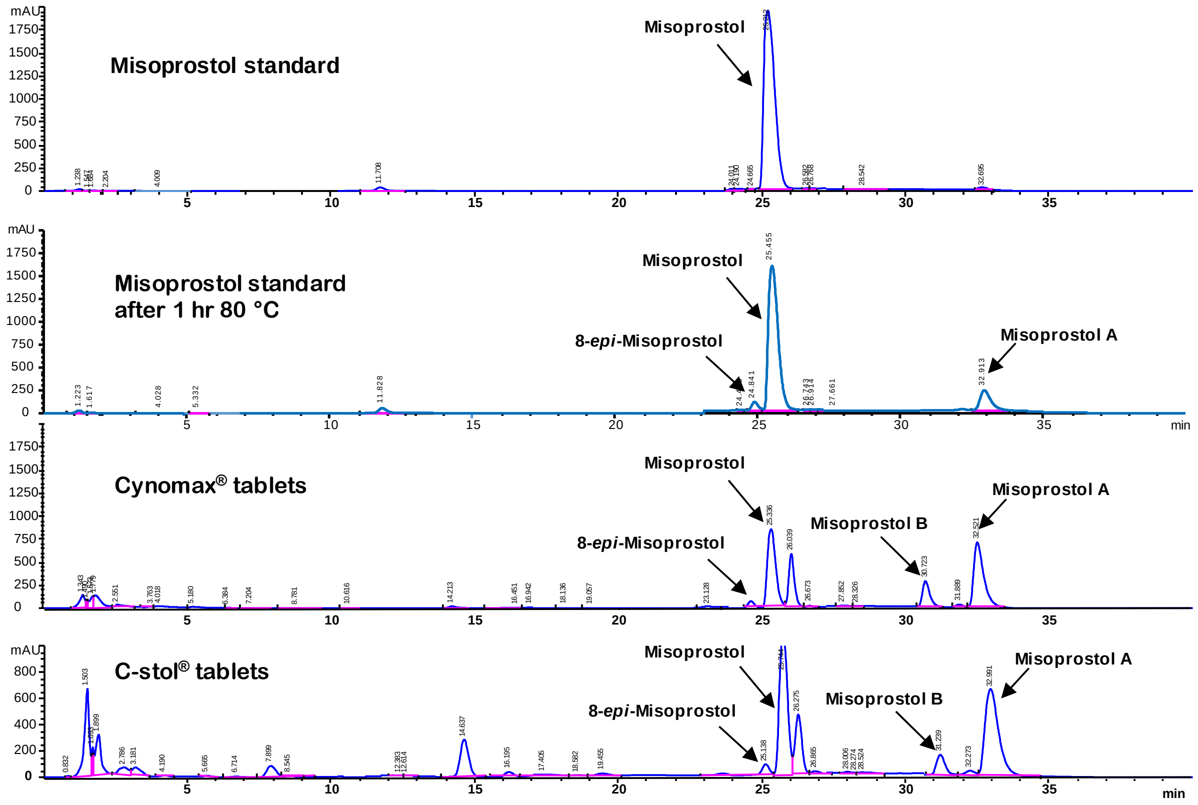

S3 Fig: HPLC analysis for related substances in two misoprostol samples.

Cynomax® tablets (batch M8TAB1801) and C-stol® tablets (batch ERW-005) were investigated. See Methods section for experimental details, and Results section for details of the investigated brands and batches.
