# Supplementary material for: Quality of oxytocin and misoprostol in health facilities of Rwanda
Source: PLoS One. 2021 Jan 8;16(1):e0245054. doi: 10.1371/journal.pone.0245054 (PMC7793248; doi:10.1371/journal.pone.0245054)
Supplement: S1 Table — (PDF) [file pone.0245054.s004.pdf]

S1 Table. List of included health facilities, number oxytocin vial and misoprostol tablets collected, and recorded oxytocin storage conditions

Facility No	Type of facility	District	Sampling site (maternity ward or storage room)	Number of collected misoprostol tablets	Misoprostol storage conditions stated on label	Number of collected oxytocin vials	Oxytocin storage conditions stated on label	Oxytocin storage site	Oxytocin storage shelf: mean kinetic temperature measured over 6 months (°C)	Refrigerator: mean kinetic temperature measured over 6 months (°C)
1	Government district hospital	Kigali city	maternity ward	50	20-25°C	10	2-8°C	refrigerator	-	**9.1**
			storage room	50; 50[e]	<30°C	10	room temperature [f]	shelf	25.3	-
2			storage room	50	<30°C	10	room temperature	shelf	24.2	-
3	Government referral hospital	Musanze	maternity ward	0	-	10	2-8°C	refrigerator	-	**8.5**
			storage room	60	<30°C	10	2-8°C	refrigerator	-	6.5
4	Faith-based district hospital	Bugesera	maternity ward	0	-	6	room temperature	shelf	26.3	-
			storage room	20	20-25°C	10	room temperature	shelf	23.4	-
5		Kamonyi	maternity ward	0	-	10	2-8°C	refrigerator	-	5.4
			storage room	51	<30°C	10	2-8°C	refrigerator	-	6.1
6		Karongi	maternity ward	0	-	10	2-8°C	cool box [a]	-	**15.8**[a]
			storage room	50	15°-25°C	10	2-8°C	refrigerator	-	not det.
7			maternity ward	10	15°-25°C	10	2-8°C	refrigerator	-	6.7
			storage room	50	<30°C	10	2-8°C	refrigerator	-	7.1
8		Muhanga	maternity ward	51	<30°C	10	room temperature	shelf	22.2	-
			storage room	50	20-25°C	10	room temperature	shelf	21.5	-
9	Government health center	Bugesera	maternity ward	0	-	5	room temperature	shelf	23.7	-
			storage room	13	20-25°C	10	room temperature	shelf	24.8	-
10			maternity ward	0	-	10	room temperature	shelf	26.0	-
			storage room	51	<30°C	10	room temperature	shelf	25.4	-
11		Kamonyi	maternity ward	0	-	8	room temperature	shelf	23.5	-
			storage room	0	-	10	room temperature	shelf	24.4	-
12			maternity ward	0	-	10	room temperature	shelf	not det.	-
			storage room	0	-	10	room temperature	shelf	25.0	-
13		Karongi	storage room	0	-	10	2-8°C	refrigerator	-	5.8
14			storage room	0	-	10	2-8°C	refrigerator	-	4.5
15		Kigali city	storage room	0	-	10	room temperature	shelf	23.7	-
16			maternity ward	0	-	10	room temperature	shelf	24.2	-
17		Muhanga	maternity ward	0	-	9	room temperature	shelf	22.3	-
			storage room	0	-	10	room temperature	shelf	21.4	-
18			storage room	0	-	10	room temperature	shelf	21.4	-
19		Musanze	storage room	0	-	10	2-8°C	refrigerator	-	7.3
20			storage room	0	-	10	2-8°C	refrigerator	-	5.7
21	Faith-based health center	Bugesera	maternity ward	0	-	10	room temperature	shelf	23.8	-
			storage room	0	-	10	room temperature	shelf	not det.	-
22			maternity ward	0	-	10	room temperature	shelf	25.1	-
			storage room	30	20-25°C	10	room temperature	shelf	**25.4** [g]	-
23		Kamonyi	storage room	0	-	10	room temperature	shelf	22.7	-
24			maternity ward	0	-	10	room temperature	shelf	22.2	-
			storage room	0	-	10	room temperature	shelf	22.1	-
25		Karongi	storage room	0	-	10	2-8°C	refrigerator	-	4.3
26			maternity ward	0	-	10	2-8°C	refrigerator	-	**-1.3**
27		Kigali city	storage room	0	-	6	2-8°C	refrigerator	-	6.5
28			storage room	0	-	10	2-8°C	refrigerator	-	**10.5**
29		Muhanga	maternity ward	0	-	9	room temperature	shelf	21.0	-
			storage room	0	-	10	room temperature	shelf	21.9	-
30			maternity ward	0	-	10	room temperature	shelf	22.7	-
			storage room	0	-	10	room temperature	shelf	22.4	-
31		Musanze	maternity ward	0	-	10	2-8°C	refrigerator	-	not det.
			storage room	0	-	10	2-8°C	refrigerator	-	5.1
32			storage room	0	-	10	2-8°C	refrigerator	-	4.7
33	Private clinic	Muhanga	storage room	0	-	10	room temperature	shelf	not det.	-
34	Retail pharmacy	Bugesera	storage room	25	no requirements	0	-	n.a.	24.1 [c]	-
35			storage room	29	no requirements	0	-	n.a.	24.0 [c]	-
36		Kigali city	storage room	50	no requirements	0	-	n.a.	24.2 [c]	-
37			storage room	28	no requirements	0	-	n.a.	23.9 [c]	-
38		Muhanga	storage room	50	no requirements	0	-	n.a.	-	-
39			storage room	48	20-25°C	0	-	n.a.	-	-
40		Musanze	storage room	50	no requirements	0	-	n.a.	-	-
41	Government central medical store	Kigali city	storage room	0	-	100;200[d,e]	2-8°C	refrigerator	-	not det.
42	Government district pharmacy	Burera	storage room	250[d]	<30°C	0	-	n.a.	-	-
43		Muhanga	storage room	200[d]	20-25°C	160[d]	room temperature	shelf	19.8	-
44	Private wholesaler	Kigali city	storage room	0	-	100[d]	room temperature	not det. [b]	-	-
45			storage room	0	-	100[d]	2-8°C	not det. [b]	-	-
46			storage room	252[d]	no requirements	100[d]	2-8°C	not det. [b]	-	-

Storage conditions which deviate from manufacturers' requirements are given in **bold, underlined** letters.
n.a. = not applicable
not det. = not determined (temperature logger not placed, lost after placement, or no data recorded).

[a] Oxytocin was stored in this maternity ward in a cool box, reportedly only ordered for immediate use.

[b] From private wholesalers, samples were purchased through a retail pharmacy using a mystery shopper approach. Therefore, storage conditions were not determined, and temperatures data loggers were not placed.

[c] In a few pharmacies, temperature loggers were placed although they did not stock oxytocin.

[d] Higher number of tablets/vials purchased as replacement samples and for additional stability testing.

[e] Two brands collected in one sampling site.

[f] Storage requirement stated on packaging: "Store in a cool dry place, away from light". Storage requirement stated on the package insert: "Store in a dark place at room temperature, protect from light."

[g] Exceeds storage temperature of misoprostol recommended by the manufacturer of the respective sample.
