# Supplementary material for: Quality of oxytocin and misoprostol in health facilities of Rwanda
Source: PLoS One. 2021 Jan 8;16(1):e0245054. doi: 10.1371/journal.pone.0245054 (PMC7793248; doi:10.1371/journal.pone.0245054)
Supplement: S2 Table — (PDF) [file pone.0245054.s005.pdf]

S2 Table. Results of chemical analysis of all oxytocin samples

Brand name and stated manu-facturer	Stated storage tempera-ture require-ment	Batch N°	Manu-facture/ Expiry Date	Faci-lity No.	Facility type	Site in facility	Mean assay (% of declared content)	RSD assay	Mean pH value	RSD pH value	Age of samples (months) [a]
Oxytocin injection; Jiangxi Xier-kangtai Pharma-ceutical Co. Ltd; China	Room tempe-rature	1606573	Jun 16/ Jun 19	2	gov. hospital	stor.	96.1% [b]	6.62% [c]	4.14	0.98%	30
				4	faith-b. hospital	mat.	102.7%	1.56%	4.03	0.48%	30
						stor.	105.5%	2.22%	4.00	0.68%	30
				8	faith-b. hospital	mat.	99.2%	0.68%	4.48	2.17	24
						stor.	99.9%	1.31%	4.40	0.96	24
				9	gov. HC	mat.	101.3%	0.55%	4.05	0.89%	30
						stor.	101.1%	1.66%	4.05	0.76%	30
				11	gov. HC	mat.	100.5%	1.73%	4.05	0.34%	30
						stor.	100.7%	1.73%	4.05	0.33%	30
				12	gov. HC	mat.	100.4%	2.29%	4.08	0.62%	30
						stor.	99.8%	1.29%	4.07	0.73%	30
				17	gov. HC	mat.	95.8%	1.71%	4.56	3.27	24
						stor.	97.9%	0.52%	4.50	1.33	24
				18	gov. HC	stor.	99.0%	0.88%	4.44	0.97	24
				23	faith-b. HC	stor.	99.1%	1.05%	4.12	0.18%	30
				24	faith-b. HC	mat.	100.4%	1.99%	4.06	0.48%	30
						stor.	95.6%	4.94%	4.19	4.38%	30
				29	faith-b. HC	mat.	98.2%	0.75%	4.46	0.73	24
						stor.	98.0%	0.79%	4.48	0.84	24
				30	faith-b. HC	mat.	99.5%	1.32%	4.38	1.18	24
						stor.	99.7%	1.18%	4.39	0.77	24
				33	private clinic	stor.	100.7%	0.96%	4.41	0.94	24
				43	distr. pharm.	stor.	101.5%	0.45%	4.06	0.32%	23
				**44**	**wholesaler**	**stor.**	**90.3% [b]**	**0.72%**	**4.08**	**0.84%**	**23**
		1604521	Apr 16/ Apr 19	1	gov. hospital	stor.	119.3%	2.09%	3.85	0.13%	32
				10	gov. HC	mat.	118.0%	1.98%	3.83	0.48%	32
						stor.	117.2%	0.52%	3.82	0.51%	32
				15	gov. HC	stor.	119.9%	3.25%	3.84	0.30%	32
				16	gov. HC	mat.	120.6%	2.24%	3.82	0.11%	32
				21	faith-b. HC	stor.	117.6%	1.02%	3.84	0.44%	32
						stor.	121.5%	1.61%	3.82	0.48%	32
				22	faith-b. HC	mat.	117.3%	1.38%	3.80	0.26%	32
						stor.	117.8%	2.31%	3.78	0.47%	32
Steroxine 10 IU/1 ml [d]; Labora-toires Sterop; Belgium	2-8°C	160042	Feb 16/ Jan 19	46	wholesaler	stor.	107.8%	0.62%	3.79	0.22%	27
		160269	Sep 16/ Aug 19	45	wholesaler	stor.	99.6%	1.11%	3.98	0.57%	20
Oxytocin 10 IU/ml; AS GRINDEKS, Latvia [e]	2-8°C	37611016	Oct 16/ Oct 20	1	gov. hospital	mat.	102.8%	1.63%	4.11	0.13%	26
				5	faith-b. hospital	mat.	102.1%	1.99%	4.09	0.15%	26
						stor.	101.9%	3.29%	4.10	0.22%	26
				20	gov. HC	stor.	99.9%	0.74%	4.11	0.10%	26
		37711116	Nov 16/ Nov 20	41	CMS	stor.	100.9%	0.82%	4.09	0.64%	18
Oxytocin 10; Rotex-medica GmbH Arznei-mittelwerk; Germany	2-8°C	70779A	Sep 17/ Sep 20	3	gov. hospital	mat.	104.1%	2.96%	4.06	0.39%	15
						stor.	102.0%	0.26%	4.02	0.20%	15
				6	faith-b. hospital	mat.	103.7%	2.09%	4.09	0.40%	15
						stor.	103.1%	1.53%	4.06	0.35%	15
				7	faith-b. hospital	mat.	103.9%	1.92%	4.02	0.10%	15
						stor.	103.8%	1.84%	4.03	0.10%	15
				13	gov. HC	stor.	103.8%	1.67%	4.02	0.19%	15
				14	gov. HC	stor.	104.2%	1.93%	4.05	0.30%	15
				19	gov. HC	stor.	102.6%	0.44%	4.00	0.10%	15
				25	faith-b. HC	stor.	103.6%	1.76%	4.02	0.24%	15
				26	faith-b. HC	mat.	103.9%	1.67%	4.01	0.19%	15
				27	faith-b. HC	stor.	105.9%	3.96%	3.97	0.19%	15
				28	faith-b. HC	stor.	105.2%	2.37%	3.99	0.21%	15
				31	faith-b. HC	mat.	103.4%	0.86%	4.00	0.14%	15
						stor.	105.4%	2.44%	3.99	0.19%	15
				32	faith-b. HC	stor.	104.6%	2.51%	4.01	0.20%	15
				41	CMS	stor.	102.4%	0.89%	3.99	0.47%	8

RSD = relative standard deviation; gov. = government; faith-b. = faith-based; HC = health center; CMS = government central medical store; stor. = storage room; mat. = maternity ward. The sample found to contain only 0.004 % benzyl alcohol is shown in **bold print**.

[a] Age of sample at time of analysis.
[b] Deviating concentration of the preservative benzyl alcohol observed, see main text.
[c] See text for explanation of the high standard deviation observed for this specific sample.
[d] Batch 160269 was labeled with the unbranded generic name "Oxytocin 10 IU/1 ml", all other information was identical as in batch 160042.
[e] Marketing authorization holder: Peckforton Pharmaceuticals Ltd., United Kingdom
