# Supplementary material for: Quality of oxytocin and misoprostol in health facilities of Rwanda
Source: PLoS One. 2021 Jan 8;16(1):e0245054. doi: 10.1371/journal.pone.0245054 (PMC7793248; doi:10.1371/journal.pone.0245054)
Supplement: S3 Table — (PDF) [file pone.0245054.s006.pdf]

S3 Table. Results of chemical analysis of all misoprostol samples

Brand name and stated manufacturer	Stated storage requirements	Batch N°	Manu-facture/ Expiry Date	Faci-lity no.	Facility type	Site in faci-lity	Mean assay (% of declared content)	RSD assay	Mean dissolution (% of declared content)	RSD disso-lution	Age of samples (months) at time of analysis
Cytotec® 200 µg, Piramal Healthcare UK Limited; United Kingdom [a]	No special storage requirements [b]	B15445 [a]	Dec 16[c] / Nov 19	38	retail pharm.	stor.	104.2%	0.13%	104.2%	0.61%	18
				40	retail pharm.	stor.	94.7%	0.27%	103.5%	2.79%	24
		B17173 [a]	Jul 17[c] / Jun 20	1	gov. hospital	stor.	95.9%	0.19%	100.1%	1.45%	17
				34	retail pharm.	stor.	94.8%	0.29%	101.2%	2.19%	17
				35	retail pharm.	stor.	94.6%	0.35%	100.1%	2.81%	17
				36	retail pharm.	stor.	94.8%	0.58%	100.8%	1.59%	17
				37	retail pharm.	stor.	95.6%	0.48%	101.5%	3.05%	17
				46	wholesaler	stor.	102.4%	1.13%	97.5%	2.82%	10
	Store at room temperature (15-25 °C)	B18097 [b]	Nov 17[c] / Oct 20	6	faith-b. hospital	stor.	95.1%	0.56%	90.6%	4.49%	13
				7	faith-b. hospital	mat.	95.5%	3.80%	102.8%	2.69%	13
Ace Miso®, Acme Formulation Pvt. Ltd.; India	Do not store above 30°C, protect from light	ACE160963	Sep 16/ Aug 18	42	gov. district pharm.	stor.	102.0%	0.44%	97.7%	2.59%	20
MIZO®, SYNOKEM Pharmaceuticals LTD; India	Store at a temperature not exceeding 30°C at a dry place	E6SGFT010	Jun 16/ May 18	8	faith-b. hospital	mat.	98.4%	0.02%	101.2%	3.51%	24
		E6SGLT004	Dec 16/ Nov 18	10	gov. HC	stor.	92.1%	1.62%	98.1%	3.18%	24
China resources, ZIZHU Pharmaceuticals Co Ltd; China	Store at a tempera-ture not exceeding 30 °C	45180301	Feb 18/ Feb 20	1	gov. hospital	stor.	95.8%	0.20%	97.7%	2.31%	10
				3	gov. hospital	stor.	94.8%	0.16%	100.1%	2.64%	10
C-stol®, CORONA Remedies Pvt Ltd; India	Store below 30°C. Protect from light and moisture	ERW-005	Mar 18/ Feb 21	2	gov. hospital	stor.	46.8%	1.33%	32.9%	15.61%	9
				5	faith-b. hospital	stor.	46.2%	0.14%	33.6%	6.82%	9
				7	faith-b. hospital	stor.	48.5%	2.96%	36.4%	4.53%	9
Cynomax®, MAXTAR BIO-GENICS; India	Store at 20° to 25°C in a dry area	M8TAB1801	May 18/ Apr 20	1	gov. hospital	mat.	42.5%	0.51%	39.8%	1.35%	7
				4	faith-b. hospital	stor.	44.6%	0.95%	39.3%	4.15%	7
				9	gov. HC	stor.	45.6%	1.07%	41.1%	3.53%	7
				22	faith-b. HC	stor.	44.9%	0.27%	41.2%	2.37%	7
		MTYX-1604	Aug 16/ Jul 18	8	faith-b. hospital	stor.	48.6%	0.06%	46.4%	3.36%	22
				39	retail pharm.	stor.	47.1%	0.04%	45.3%	1.57%	22
				43	gov. district pharm.	stor.	47.6%	0.24%	47.6%	4.49%	21

RSD = relative standard deviation
gov. = government
faith-b. = faith-based
HC = health center
pharm. = pharmacy
stor. = storage room
mat. = maternity ward.

[a] Marketing authorization holder: Pfizer Holding, France.

[b] Marketing authorization holder: Continental Pharma Inc., Belgium.

[c] Package insert: "Tenir hors de la vue et de la portée des enfants. Pas de précaution particulière de conservation". I.e.: "Keep out of sight and reach of children. No special storage requirements."

[d] Manufacturing date not stated on packaging. Shelf-life listed according to information from the websites www.hpra.ie and www.medicines.org.uk/emc.
